# Safety and Adverse Events Among Long-term Care Residents Receiving a Third COVID-19 mRNA Vaccine Booster Dose in Quebec

**DOI:** 10.1001/jamanetworkopen.2022.23401

**Published:** 2022-07-21

**Authors:** Xi Sophie Zhang, Andréanne Moreau, Diana Cruz-Santiago, Stéphanie Langevin, Quoc Dinh Nguyen

**Affiliations:** 1 Department of General Medicine, Centre intégré de santé et de services sociaux du Centre-Sud-de-l’Île-de-Montréal, Montréal, Quebec, Canada; 2 Department of Family and Emergency Medicine, Université de Montréal, Montréal, Quebec, Canada; 3 Division of Family Medicine, Department of Geriatrics, Centre intégré de santé et de services sociaux du Centre-Sud-de-l’Île-de-Montréal, Montréal, Quebec, Canada; 4 Centre de recherche de l’Institut de gériatrie de Montréal, Montréal, Quebec, Canada; 5 Department of Specialty Medicine, Centre intégré de santé et de services sociaux du Centre-Sud-de-l’Île-de-Montréal, Montréal, Quebec, Canada; 6 Department of Microbiology, Infectious Disease, and Immunology, Université de Montréal, Montréal, Quebec, Canada; 7 Division of Geriatrics, Department of Medicine, Centre hospitalier de l’Université de Montréal, Montréal, Quebec, Canada; 8 Centre de recherche du Centre hospitalier de l’Université de Montréal, Montréal, Quebec, Canada; 9 Department of Medicine, Université de Montréal, Montréal, Quebec, Canada

## Abstract

This cohort study examines the safety and adverse events associated with receipt of various 3-dose combinations of COVID-19 mRNA vaccines with among long-term care residents in Quebec, Canada.

## Introduction

Booster doses of mRNA COVID-19 vaccines are administered worldwide to enhance antibody levels and immunity against Delta, Omicron, and emerging variants. Data on adverse events show a favorable safety profile, with similar reactogenicity for second and third doses. However, safety in older adults in long-term care (LTC) settings has not been well described.^1,2^ We examined adverse events following mRNA booster vaccination in LTC residents within the context of factors unique to the province of Quebec that may increase reactogenicity: extended interval between doses 1 and 2,^3^ heterologous vaccination, full dose mRNA-1273 (100 µg) booster,^4^ and high prevalence of previous COVID-19 infection.^5^

## Methods

This cohort study was approved by the Centre-Sud-de-l’Île-de-Montréal regional health authority (CS-RHA) institutional review board. We followed the STROBE reporting guideline.

We included 280 LTC residents from 6 facilities of the CS-RHA in Quebec. Residents received mRNA booster doses, BNT162b2 (Pfizer [P]), 30 µg, or mRNA-1273 (Moderna [M]), 100 µg, between October 18 and November 1, 2021. In Quebec, a minimal interval of 3 months between doses 1 and 2 was used. The 100-µg mRNA-1273 dosage rollout was planned before the 50-µg booster dose FDA approval. Residents were initially eligible regardless of prior COVID-19 infection status and vaccination combination for the first 2 doses (any combination of BNT162b2 and mRNA-1273), 5 months after the second dose. We excluded residents who received the second dose less than 35 days after the first dose (n = 10) and those who received a third dose for indications related to immunosuppression. We collected age, sex, COVID-19 infection date, vaccination combinations, and dates. The primary outcome was the occurrence of any systemic adverse events (SAEs, ie, fever, malaise, gastrointestinal symptoms, tachycardia, desaturation, respiratory distress, hospital transfer, hypotension, thrombosis, rigors, death), during the first 48 hours. SAEs were collected as part of instructed postvaccination surveillance by health care staff and reported in the medical record. We calculated the proportion of residents reporting SAEs for vaccine combinations with more than 40 participants. To assess factors of reactogenicity, we used logistic regression with vaccine combinations and COVID-19 status as independent variables, adjusted for age (b-splines) and sex. The level of statistical significance was α = .05 (2-sided); analyses were conducted using R version 4.1.2 (R Project for Statistical Computing).

## Results

Among 280 participants included, 184 (66%) were female and 96 (34%) were male; the mean (SD) age was 83 (11) years. The Table reports the demographic characteristics by vaccine combinations (MPM, MPP, PPP). The median (IQR) interval between doses 1 and 2 was 111 (1) days and between 2 and 3 was 175 (1) days; the interval between COVID-19 infection and dose 1 was 266 (25) days. Figure A compares SAEs for the MPM combination according to prior COVID-19 infection (+). Figure B, C, and D present results in those without prior infection (−) for MPM, MMP, and PPP. Among residents with MPM+, 46% had a SAE (40% fever) compared with 16% (10% fever) for MPM−, 6% for MMP−, and 2% for PPP−. With the MPM− as reference, the likelihood of SAEs was greater for MPM+ (OR, 4.13; 95% CI, 2.08-8.47) and lower for MPP− (OR, 0.27; 95% CI, 0.06-0.90) and PPP− (OR, 0.12; 95% CI, 0.01-0.63). No resident had a COVID-19 infection within 7 days of vaccination.

**Table.** Demographic Characteristics of Long-term Care Residents and Vaccination Intervals by Vaccine Combination and Prior COVID-19 Infection

Characteristics	Overall	MPM COVID-19	MPM	MMP	PPP
Residents, No.	280	79	104	52	45
Age, mean (SD), y	83 (11)	85 (10)	85 (11)	80 (13)	79 (11)
Sex, No. (%)					
Female	184 (66)	59 (75)	69 (66)	33 (63)	23 (51)
Male	96 (34)	20 (25)	35 (34)	19 (37)	22 (49)
Prior COVID-19 infection, No. (%)	79 (28)	79 (100)	0	0	0
Interval, median (IQR), d					
Between infection and dose 1^a^	NA	266 (25)	NA	NA	NA
Between dose 1 and 2	111 (1)	111 (1)	111 (1)	112 (0)	95 (0)
Between dose 2 and 3	175 (1)	175 (1)	176 (1)	174 (0)	190 (3)

Abbreviations: M, mRNA-1273; NA, not applicable; P, BNT-162b2.

^a^ One COVID-19 infection occurred between doses 1 and 2.

**Figure.**
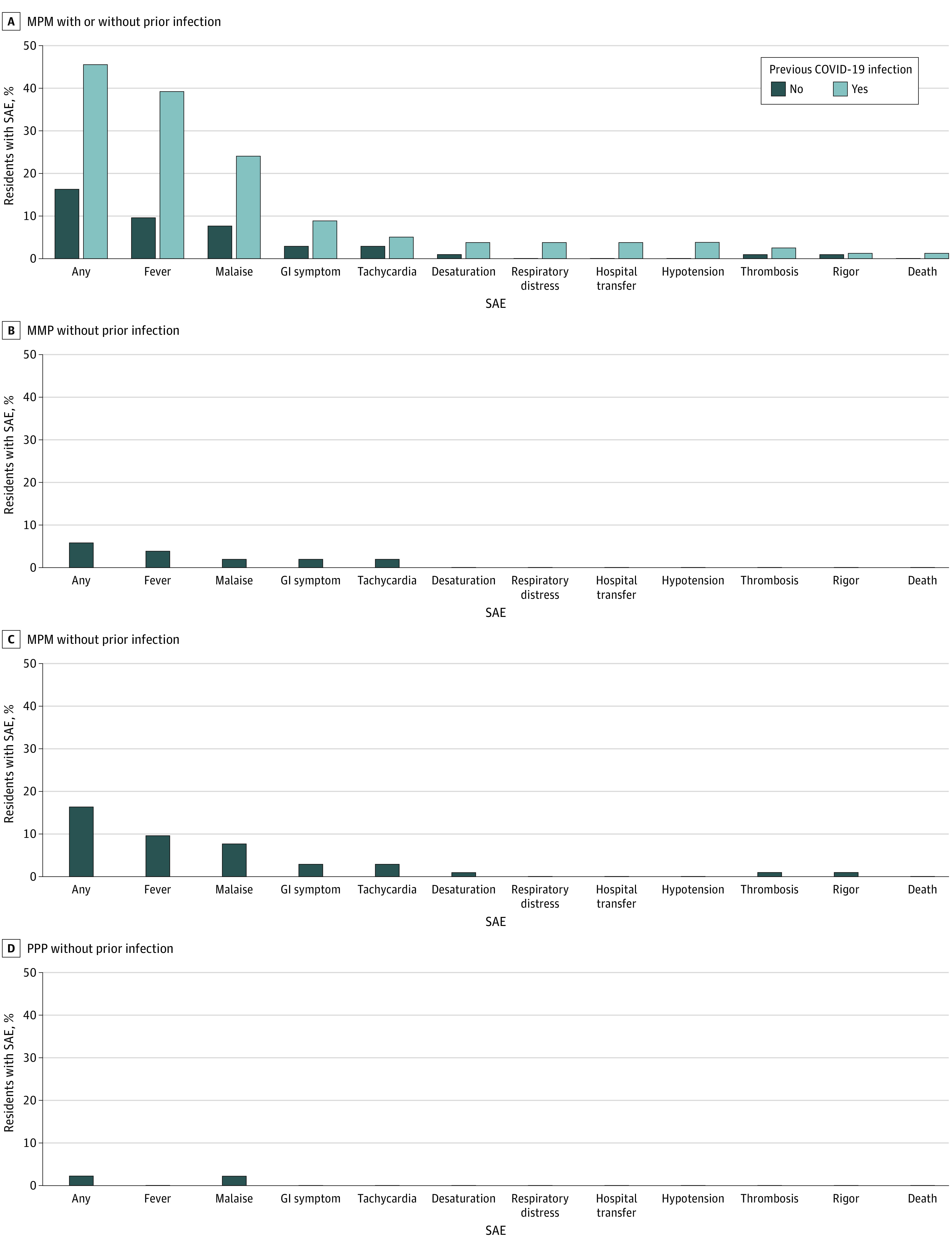
SAEs and mRNA Vaccination Combination Among Those With and Without Prior COVID-19 Infection GI indicates gastrointestinal; M, mRNA-1273; P, BNT-162b2; SAE, systemic adverse event.

## Discussion

In comparison with previous reports of third doses’ reactogenicity,^2,4^ our findings indicate a high proportion of systemic adverse events, specifically among LTC residents with prior infection. SAEs were also more likely with MPM compared with MPP and PPP, suggesting that the mRNA-1273 100-µg booster dose and heterologous vaccination may increase reactogenicity.^6^ The extended interval between doses 1 and 2 may have increased immunogenicity.^3^ Although LTC residents are disproportionately at risk of severe outcomes following COVID-19 infection, our findings suggest that they may also be at greater risk of postvaccination adverse effects. As additional booster doses are considered due to waning immunity and variants, examination of past vaccines and intervals, immunity status, and booster dosage may be required to weigh their potential benefits against the risk of adverse effects.

Limitations of our study include absence of antibody testing for prior infections, infections prior to the Omicron variant, and no direct comparison of SAEs between the third and previous doses. Our results may not be generalizable to other older adult populations.
